# Supplementary material for: Quantitative basis of meiotic chromosome synapsis analyzed by electron tomography
Source: Sci Rep. 2019 Nov 6;9:16102. doi: 10.1038/s41598-019-52455-4 (PMC6834585; doi:10.1038/s41598-019-52455-4)
Supplement: Supplementary file 1 — Supplementary Information [file 41598_2019_52455_MOESM1_ESM.pdf]

Quantitative basis of meiotic chromosome synapsis analyzed by electron tomography

Marie-Christin Spindler[1], Sebastian Filbeck[1], Christian Stigloher[2], Ricardo Benavente[1,*]

[1]*Department of Cell and Developmental Biology, Biocenter, University of Würzburg, 97074 Würzburg, Germany*

[2]*Imaging Core Facility, Biocenter, University of Würzburg, 97074 Würzburg, Germany*

[3]*Center for Computational and Theoretical Biology, University of Würzburg, 97074 Würzburg, Germany*

Supporting tables

initial temperature	final temperature	duration	solution	comment
-90 °C		96 h	0.5 % glutaraldehyde + 0.1 % tannic acid in anhydrous acetone	exchange solution once
-90 °C		4-6 h	anhydrous acetone	4 consecutive wash steps
-90 °C		28 h	2% OsO4 in anhydrous acetone	
-90 °C	-20 °C	14 h		
-20 °C		16 h		
-20 °C	4 °C	4 h		
4 °C		2-3 h	anhydrous acetone	4 consecutive wash steps
4°C	RT			remove sample pellet from carrier
RT		5 h	50 % epoxy resin in acetone	
4 °C		over night	90 % epoxy resin in acetone	
RT		2-3 h	100 % epoxy resin	
RT		2-3 h	100 % epoxy resin	
RT		2-3 h	100 % epoxy resin	
RT			100 % epoxy resin	polymerisation for at least 48 h at 60 °C

Supplementary Table 1. Freeze substitution protocol. Also published in ”Spindler, MC., Redolfi, J., Helmprobst, F., Kollmannsberger, P., Stigloher, C. & Benavente, R. Electron tomography of mouse LINC complexes at meiotic telomere attachment sites with and without microtubules. *Communications Biology* **2**:376 (2019).”

Metric	Significant Difference	p-value (Wilcoxon rank-sum)
Length	No	0.1156
LE-Indent	No	0.5881
CE-Indent	No	0.2327
MinDistance	No	0.0902
CR-Width	No	0.2217

Supplementary Table 2. Statistical tests for significant differences between the transverse filament length, indentation in the lateral element (LE-indent), indentation in the central element (CE-indent), their minimum distance (MinDistance) as well as the width of the central region (CR-width) in tomograms of SCs at attachment sites and tomograms of interstitial SCs.

Supporting figures

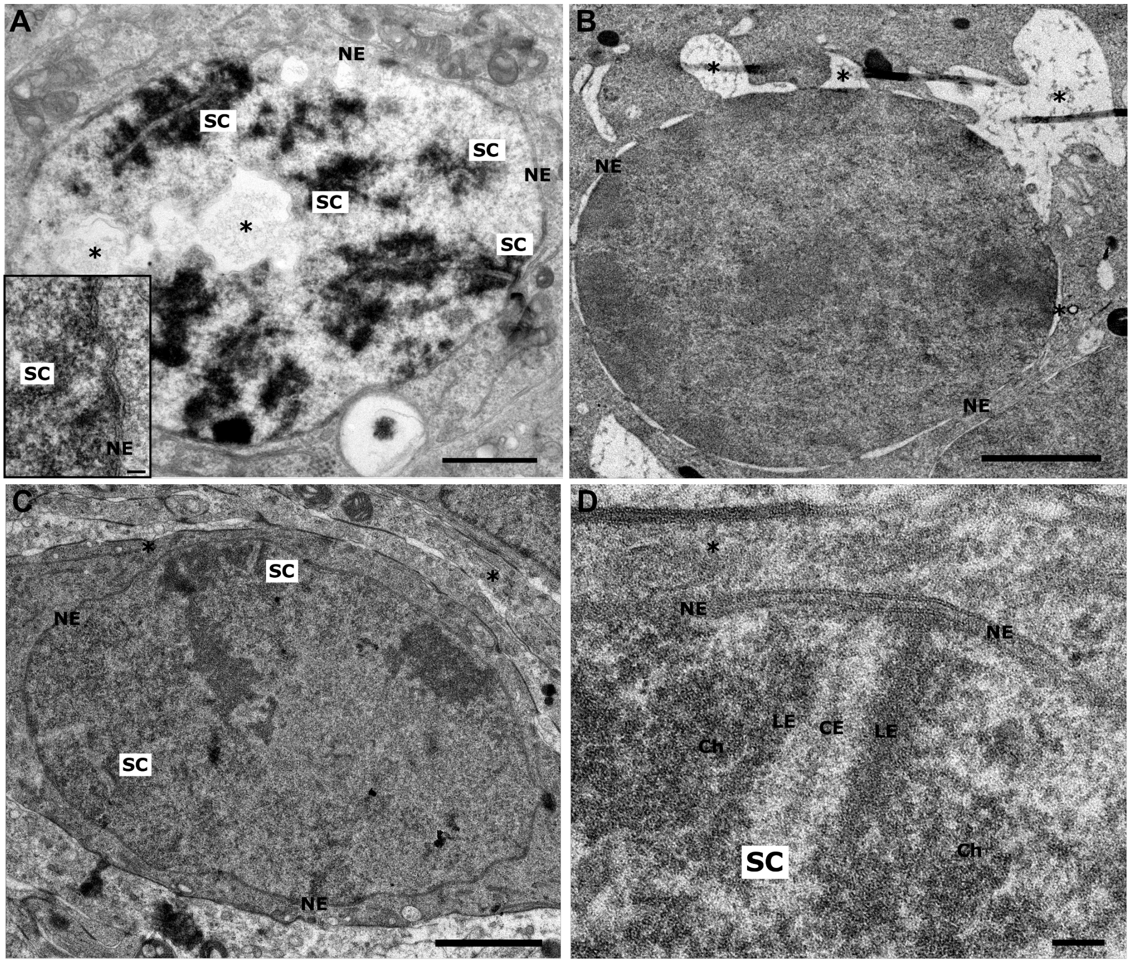

Fig. S 1. Ultrastructural preservation in pachytene spermatocytes. Structural preservation after chemical fixation (A), high-pressure freezing followed by freeze substitution (B) and pre-fixation with subsequent high-pressure freezing and freeze substitution (C,D). A: Dehydration at room temperature introduces shrinking artefacts. These artefacts are particularly noticeable at the nuclear envelope where they are characterized by a variation in perinuclear space as shown at a telomere attachment site of chemically frozen tissue (inset). Asterisks indicate additional artefacts caused by poor resin infiltration and polymerization. B: In the high-pressure frozen spermatocyte, the nuclear content is detached from the nuclear envelope due to shearing forces during preparation and high-pressure freezing (asterisks). The chromatin here is affected by a slight segregation pattern caused by ice crystal formation. C, D: Good tissue and ultrastructural preservation of pachytene spermatocyte (C) and corresponding telomere attachment site (D) after chemical fixation combined with high-pressure freezing and freeze substitution. The spermatocyte appears far less extracted and at the telomere attachment site, the nuclear envelope has a continuous spacing. SC: synaptonemal complex; NE: nuclear envelope; LE: lateral element, CE: central element; Ch: Chromatin. Scale bars: 2 μm (A-C); 100 nm (D).

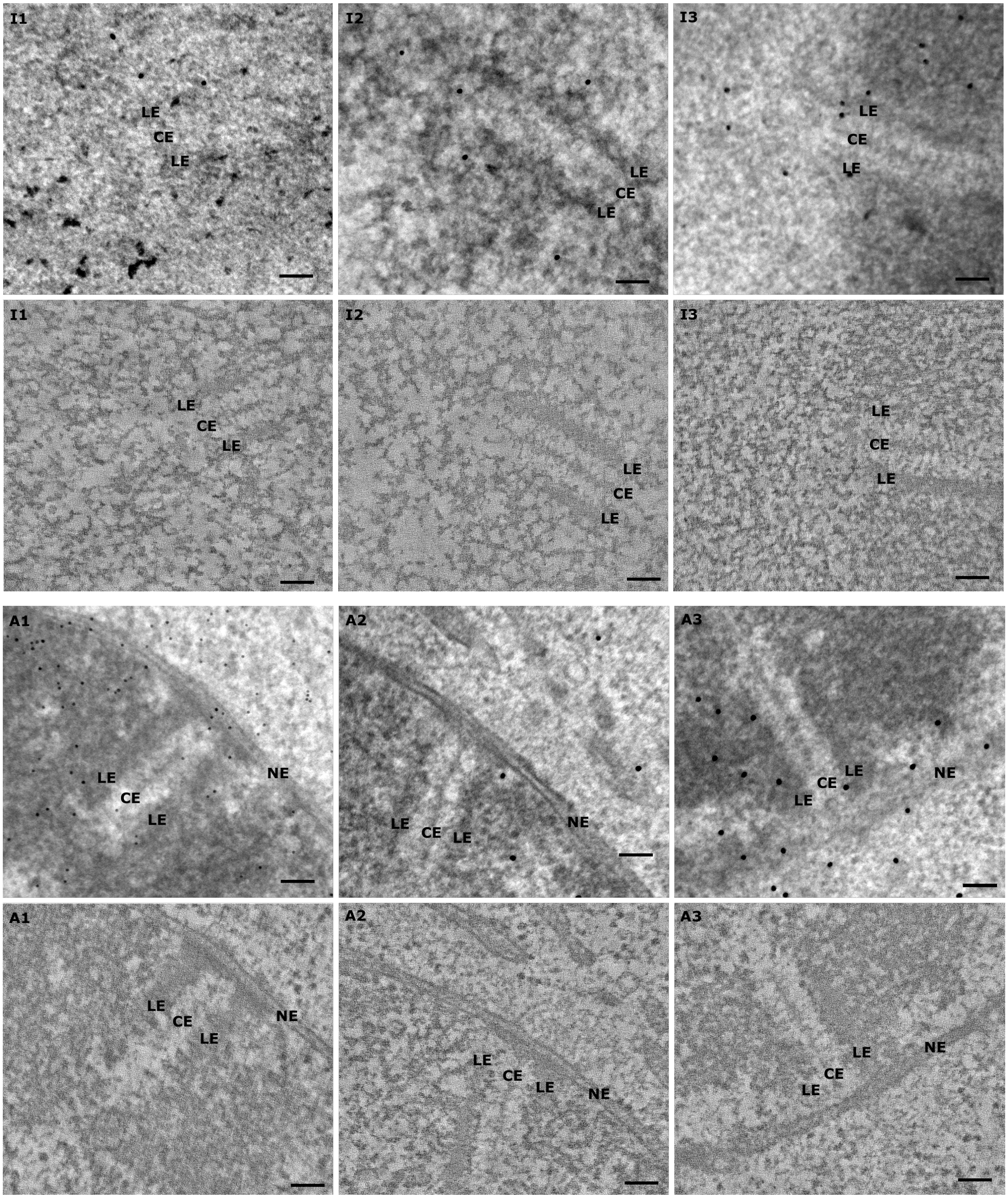

Fig. S 2. Orientation of analyzed synaptonemal complexes. For each of the 6 tomograms containing the synaptonemal complexes analyzed in this study, a TEM tilted image acquired at 0° tilt and a central virtual slice of the reconstructed tomogram are shown one below the other. Top panel: interstitial synaptonemal complexes I1-3), i.e. SCs in the center of the spermatocyte; Bottom panel: attached synaptonemal complexes (A1-3), i.e. SCs attached to the nuclear envelope. LE: lateral element; CE: central element; NE: nuclear envelope. Scale bar: 100 nm.

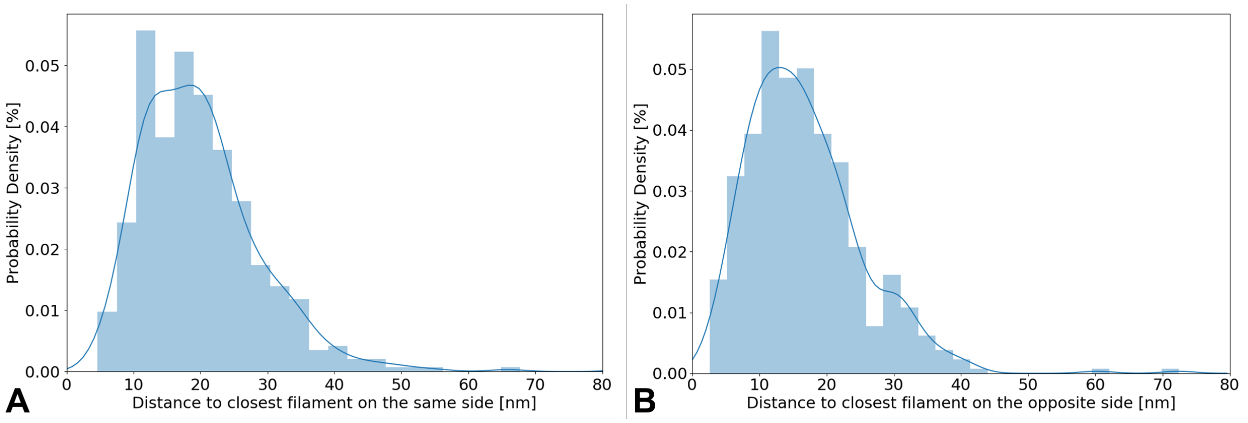

Fig. S 3. Distribution plots of the CE-endpoint distance to the CE-endpoint of the closest opposing/ parallel transverse filament (TF).
